# Identifying and analyzing high-prescribers of opioids and antibiotics using medicare part D data

**DOI:** 10.1017/ash.2025.10285

**Published:** 2026-02-13

**Authors:** Amulya Marellapudi, Raysha Farah, Justin Halim, Prabhu Sasankan, Priya Nori

**Affiliations:** 1 Harvard T.H. Chan School of Public Health, Boston, MA, USA; 2 Department of Medicine, Montefiore Health System, Albert Einstein College of Medicine, Bronx, NY, USA; 3 Department of Medicine, Division of Infectious Diseases, Montefiore Health System, Albert Einstein College of Medicine, Bronx, NY, USA; 4 Beth Israel Deaconess Medical Center/Harvard Medical School, Boston, MA, USA

## Abstract

**Background:**

Excess prescribing of antibiotics and opioids is a major public health concern. A greater understanding of prescribing patterns at the prescriber and beneficiary level could inform enhanced and integrated interventions.

**Methods:**

Using 2023 Medicare Part D Public Use data, we conducted a cross-sectional study to assess opioid and antibiotic prescribing patterns among primary care clinicians (internal medicine, family medicine, geriatrics, nurse practitioners, and physician assistants) in New York State (NYS) treating ≥20 Medicare beneficiaries aged ≥65. For each provider, the total days’ supply of antibiotics and opioids per beneficiary was calculated. Multivariate logistic regression models identified provider and practice characteristics associated with high-prescribing.

**Results:**

Of the 19,823 eligible prescribers, 647 (3.3%) were high antibiotic prescribers and 554 (2.8%) were high opioid prescribers. Antibiotic high-prescribing was associated with nurse practitioners (NP) (adjusted odds ratio (aOR 2.47, 95% CI 1.83–3.34)), physician assistants (PA) (aOR 1.90, 95% CI 1.43–2.54), more years in practice (aOR 1.62 per SD, 95% CI 1.47–1.78), and panels with higher average beneficiary risk scores (aOR 1.36 per SD, 95% CI 1.29–1.43). Opioid high-prescribing was associated with geriatrics (aOR 4.30, 95% CI 1.79–10.32), NP (aOR 1.80, 95% CI 1.36–2.37), male gender (aOR 1.55, 95% CI 1.17–2.04).), greater years in practice (aOR 1.68, 95% CI 1.51–1.86), and higher proportions of dual-eligible beneficiaries (aOR 1.38, 95% CI 1.23–1.56).

**Conclusions:**

A small number of NYS clinicians account for a disproportionate share of antibiotic and opioid prescribing. Identifying provider- and panel-level characteristics associated with higher prescribing may inform targeted stewardship strategies.

## Introduction

Antibiotic overprescribing remains a global concern with antibiotic prescriptions representing more than 40% of all outpatient primary care prescriptions.^1^ In 2022, outpatient providers in the United States issued approximately 236 million antibiotic prescriptions.^2^ Between 2021 and 2022 opioids totaled nearly 70 million outpatient prescription fills.^3^ Inappropriate prescribing of both antibiotics and opioids remains a critical public health issue due to antimicrobial resistance (AMR), overdose-related mortality, increased healthcare costs, and exacerbated health disparities – especially among Medicare populations.^4–5^

A recent CDC analysis from 2019 Medicare Part D data revealed that 10% of prescribers accounted for nearly 41% of outpatient antibiotic prescriptions, underscoring the concentration of prescribing volume among a small subset of clinicians.^6^ Despite ongoing efforts like the CDC’s *Core Elements of Outpatient Antibiotic Stewardship and the 2022 update on the* Clinical Practice Guideline for Prescribing Opioids for Pain, little is known about provider- and practice-level factors linked to high prescribing of antibiotics, opioids, or both.^7–10^

A recent analysis of dental and primary care clinicians – which classified “high prescribers” as those in the top 25% of prescribing volume – likewise found that older provider age, geographic region, and high prescribing in one drug class were associated with high prescribing in the other.^4^ Similarly, a study based in Washington state revealed that high antibiotic prescribing is a function of older age, male sex, and high opioid prescribing behavior.^11^ Identifying characteristics of high-volume prescribers has informed targeted interventions, like antibiotic prescribing peer-comparison report cards and opioid stewardship programs, which have reduced overall prescribing.^8,11,12^

In this study, using 2023 Medicare Part D data for New York State primary care providers, we sought to: (1) measure the prevalence and overlap of dual high prescribing; (2) identify provider-level and practice-level characteristics associated with high prescribing of opioids, antibiotics, or both; (3) inform targeted intervention strategies to address dual high prescribing.

## Methods

We conducted a cross-sectional analysis of opioid and antibiotic prescriptions using the 2023 Centers for Medicare and Medicaid Services (CMS) Part D Prescriber Public Use Files. The study cohort included all New York State prescribers (physicians, nurse practitioners, and physician assistants) in primary care specialties – Internal Medicine, Family Medicine, and Geriatrics – who treated at least 20 Medicare beneficiaries aged 65 years or older. Provider records were linked to the National Plan and Provider Enumeration System (NPPES) to obtain gender and years in practice.

Drug classifications for oral antibiotics and opioids were derived from the Medicare Part D drug reference list and verified by the study team for accuracy. Outpatient intravenous (IV) antibiotics and opioids were excluded because these medications are generally administered in infusion settings under specialist supervision and are not dispensed through outpatient retail pharmacies. Topical, otic, and ophthalmic antibiotics were also excluded. For each provider, we calculated the total days’ supply for antibiotics and opioids, divided by the number of beneficiaries aged ≥65 years, consistent with CDC methodologies.^6,7^ High-prescribing providers were defined as those in the top 10% of this distribution for each drug class.^6^

A multivariate logistic regression analysis was conducted to understand the specific characteristics of providers and practice settings associated with high prescribing. High-prescribing status (physicians in the top decile for antibiotics or opioids) was modeled as a binary dependent variable. These variables included provider specialty, gender, years in practice (from the National Provider Identifier enumeration date), total claims per beneficiary, Rural–Urban Commuting Area (RUCA) code category, and beneficiary panel characteristics (average age, risk score based on the health status and demographics of enrollees, proportion dual-eligible for Medicare and Medicaid, and racial/ethnic composition). Covariates were selected based on published literature demonstrating their association with outpatient antibiotic and opioid prescribing behaviors.^7,11^ Country or state where provider trained was not available in the Part D data set and therefore could not be included as a covariate in our analysis. RUCA codes are defined as metropolitan (codes 1–3), micropolitan (codes 4–6), small towns (codes 7–9), rural areas (code 10). All analyses were conducted in Python version 3.11.

## Outcomes and predictors

The primary outcomes were binary indicators of high antibiotic and high opioid prescribing, defined as total days’ supply per beneficiary, calculated separately for each drug class among providers with at least one claim. High-prescribing providers were those in the top decile of this distribution for their respective drug class.

Provider-level characteristics included specialty, gender, and number of years in practice. Beneficiary panel characteristics consisted of average beneficiary age, average Hierarchical Condition Category (HCC) risk score, proportions of beneficiaries who were dual-eligible for Medicare and Medicaid, and the distribution of beneficiaries by race/ethnicity (White, Black, Hispanic, Asian/Pacific Islander, American Indian/Native Alaskan). The HCC score is a risk-adjustment tool developed by CMS to predict future healthcare costs for Medicare beneficiaries based on their demographics and diagnosed medical conditions. Dual eligibility serves as a marker of socioeconomic inequities, identifying beneficiaries with greater medical and social needs. Racial and ethnic composition of beneficiaries was considered separately to account for potential differences in access and prescribing patterns.

Using these factors, a predictive model was created to identify key risk factors using a five-fold logistic regression. Five-fold cross-validation was used to evaluate model performance. The data set was divided into five equal subsets, with the model trained on four subsets and validated on the fifth in rotation. Continuous predictors were standardized, and categorical variables were encoded as binary indicators. Model performance was evaluated using the area under the receiver operating characteristic curve (AUROC) and model calibration was evaluated using the Brier score, defined as the mean squared error between predicted probabilities and binary outcomes. Adjusted odds ratios (ORs) and 95% confidence intervals (CIs) were derived after refitting the model to the full data set. Family medicine served as the reference group (OR = 1.0); all odds ratios were reported relative to this group.

## Results

### Overall characteristics

Among 19,905 primary care prescribers, 647 (3.3%) were high antibiotic prescribers and 554 (2.8%) were high opioid prescribers. Only 51 (.3% of all prescribers) were high for both antibiotics and opioids, representing 7.9% of high antibiotic prescribers and 9.2% of high opioid prescribers. Dual high prescribers were therefore rare, and analyses focused on high antibiotic and high opioid prescribers separately. The most frequently prescribed antibiotic classes were penicillins, cephalosporins, macrolides, and fluoroquinolones. Among opioids, the most frequently prescribed agents were hydrocodone–acetaminophen, oxycodone, tramadol, and codeine-containing formulations.

For both opioids and antibiotics, prescribing volume was highly concentrated among a small subset of clinicians. Among primary care prescribers, providers in the top decile for antibiotic volume accounted for 77.1% of total days of antibiotics supplied; meanwhile, those in the top decile for opioid volume accounted for 83.7% of all opioid days of supplied. 5-fold cross-validation, discrimination was acceptable for both outcomes. For high antibiotic prescribing, the AUROC was .694 (95% CI .674–.715; Brier score .031). For high opioid prescribing, the AUROC was .754 (95% CI .735–.774; Brier score .026).

### Antibiotic prescribing (Figure 1):

In the multivariate logistic regression, high antibiotic prescribing was more common among physician assistants (PA) (OR 1.90, 95% CI 1.43–2.54) and nurse practitioners (NP) (OR 2.47, 95% CI 1.83–3.34) compared with family medicine physicians. Internal medicine physicians similarly had greater odds of high prescribing (OR 1.55, 95% CI 1.17–2.04). Among continuous predictors, each one–standard deviation increase in years in practice (SD ≈ 5.78 yr) was associated with higher odds of being a high antibiotic prescriber (aOR 1.62 per SD, 95% CI 1.47–1.78), as was a one–standard deviation increase in average beneficiary risk score (aOR 1.36 per SD, 95% CI 1.29–1.43). Panels with a greater proportion of white beneficiaries (OR 1.33 per SD, 95% CI 1.17–1.50) showed higher odds. In contrast, panels with a higher proportion of black beneficiaries had significantly lower odds (OR .81 per SD, 95% CI .73–.90). Male provider gender was also positively associated (OR 1.27, 95% CI 1.06–1.52) with high antibiotic prescribing.

**Figure 1.**
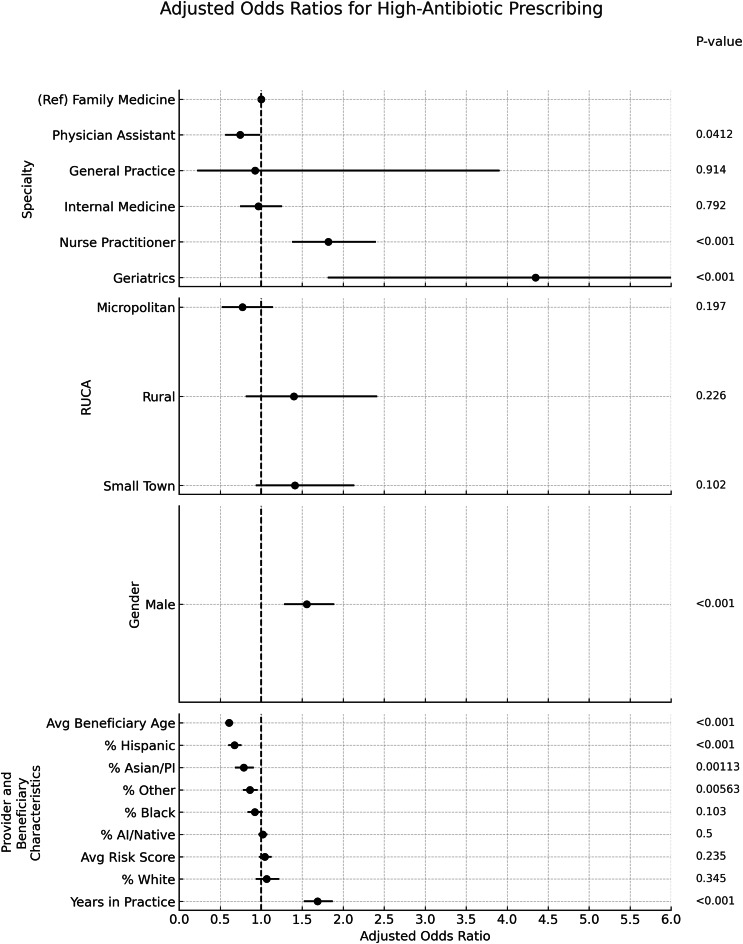
Adjusted odds ratios (ORs) and 95% confidence intervals for predictors of high antibtioic prescribing.

### Opioid prescribing (Figure 2):

For high opioid prescribing, geriatrics had the strongest association (OR 4.30, 95% CI 1.79–10.32) compared with family medicine (reference group). NP (OR 1.80, 95% CI 1.36–2.37) also had greater odds, while PA had lower odds (OR .74, 95% CI .56–.98). More years in practice was consistently associated with higher odds of high opioid prescribing; each one–standard deviation increase in years in practice (SD ≈ 5.78 yr) corresponded to an adjusted OR of 1.68 (95% CI 1.51–1.86), similar in magnitude to the association seen for high antibiotic prescribing. Providers treating panels with a higher share of dual-eligible beneficiaries were more likely to be high opioid prescribers (OR 1.38 per SD, 95% CI 1.23–1.56). A higher average beneficiary age was inversely associated with opioid prescribing (OR .61 per SD, 95% CI .56–.66). Lastly, being a male provider was associated with higher odds of opioid prescribing (OR 1.55, 95% CI 1.28–1.89).

**Figure 2.**
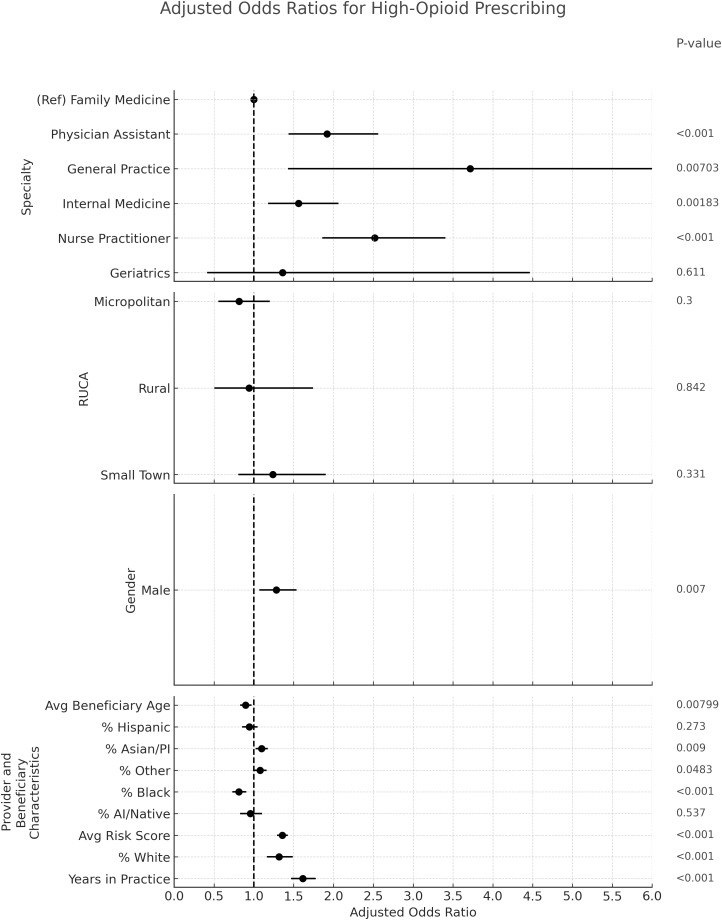
Adjusted odds ratios and 95% confidence intervals for predictors of high opioid prescribing.

## Discussion

In this cross-sectional analysis of 2023 Medicare Part D prescribing data from New York State primary care clinicians, we identified disparate but overlapping predictors of high antibiotic and opioid prescribing. Although care delivery had largely stabilized by 2023, outpatient utilization and respiratory infection trends may still have differed from prepandemic years and could have affected prescribing behavior. High-prescribers – defined as those in the top decile for days of drug supplied per beneficiary – represented a small minority of providers but, importantly, accounted for a significant share of prescribing volume.

For antibiotics, high prescribing was most strongly associated with NP and PA compared with family medicine physicians (reference group, OR = 1), greater years in practice, higher average patient risk scores, and a greater proportion of white beneficiaries. These patterns may be suggestive of differences in training, scope of practice, or patient care settings.^12,13^

For opioids, geriatrics had the strongest association, followed by NP providers, greater years in practice, and male provider gender. The strong association with geriatrics likely reflects a combination of higher chronic pain burden, palliative care needs, and multiple comorbidities among older adults.^14–15^ Interestingly, average beneficiary age was inversely associated with high opioid prescribing, likely due to concern for adverse effects in older patients.

Limitations of this study include the inability to evaluate whether prescriptions were appropriate to clinical context and reason for visit to primary care, as ICD-10 codes associated with prescriptions were not analyzed. For instance, primary care practices seeing a greater volume of visits for infectious conditions (eg, respiratory infection, cellulitis, etc.) may have a greater antibiotic prescribing volume, which is not reflected in our analysis.^16^ In addition, the limited number of geriatricians reduces statistical power and limits the generalizability of findings for this group. Prescribers participating in Medicare Part D may differ from those serving non-Medicare populations. Additionally, claims-based data are vulnerable to detection bias because clinical indications and contextual factors that influence prescribing may be incompletely captured. Lastly, unmeasured training differences including location, and exposure to education on antibiotic and opioid stewardship could influence prescribing behavior.

Furthermore, restriction to Medicare beneficiaries in a single state, and potential confounding from unmeasured patient or provider characteristics may exist. Unlike a prior Washington State analysis, this study did not identify a strong association between high opioid and high antibiotic prescribing which may reflect state-level differences in stewardship practices, patient populations, and healthcare access.^11^ In conclusion, this work highlights provider-level factors associated with higher antibiotic and opioid prescribing.

Future research should explore the behavioral and healthcare access drivers of opioid and antibiotic prescriptions and quantify prescription volume as a function of specific diagnoses as a surrogate of appropriateness. Although days’ supply provides a useful volume-based measure, it does not incorporate factors such as clinical context, or spectrum of antibiotic therapy. Future research should address these elements through integration of EHR-based prescribing data or datasets containing diagnostic indications. Importantly, future studies should specifically examine prescribing patterns among specialists such as dentists and podiatrists to inform targeted stewardship strategies. Ongoing research should also evaluate whether targeting other percentile cutoffs (eg, top 15th or 20th) may yield greater population-level impact. By identifying high-prescribers and implementing targeted interventions, healthcare organizations and policymakers can help reduce overprescribing and promote patient safety.
